# Radial Glial Dependent and Independent Dynamics of Interneuronal Migration in the Developing Cerebral Cortex

**DOI:** 10.1371/journal.pone.0000794

**Published:** 2007-08-29

**Authors:** Yukako Yokota, H.T. Ghashghaei, Christine Han, Hannah Watson, Kenneth J. Campbell, E.S. Anton

**Affiliations:** 1 UNC Neuroscience Center and the Department of Cell and Molecular Physiology, University of North Carolina School of Medicine, Chapel Hill, North Carolina, United States of America; 2 Developmental Biology Program, Cincinnati Children's Hospital, Cincinnati, Ohio, United States of America; University of Sydney, Australia

## Abstract

Interneurons originating from the ganglionic eminence migrate tangentially into the developing cerebral wall as they navigate to their distinct positions in the cerebral cortex. Compromised connectivity and differentiation of interneurons are thought to be an underlying cause in the emergence of neurodevelopmental disorders such as schizophrenia. Previously, it was suggested that tangential migration of interneurons occurs in a radial glia independent manner. Here, using simultaneous imaging of genetically defined populations of interneurons and radial glia, we demonstrate that dynamic interactions with radial glia can potentially influence the trajectory of interneuronal migration and thus the positioning of interneurons in cerebral cortex. Furthermore, there is extensive local interneuronal migration in tangential direction opposite to that of pallial orientation (i.e., in a medial to lateral direction from cortex to ganglionic eminence) all across the cerebral wall. This counter migration of interneurons may be essential to locally position interneurons once they invade the developing cerebral wall from the ganglionic eminence. Together, these observations suggest that interactions with radial glial scaffold and localized migration within the expanding cerebral wall may play essential roles in the guidance and placement of interneurons in the developing cerebral cortex.

## Introduction

Different classes of neurons of the cerebral cortex arrive at their laminar and areal locations using distinct patterns of migration. Pyramidal cortical neurons migrate radially from their birth places in the dorsal telencephalic ventricular zone, primarily by using the radial glial scaffold or the inherited leading process of a radial glial parent cell, as a template for their oriented migration [1]–[4]. Interneurons, arising from the ganglionic eminence (GE), migrate tangentially into the developing cerebral wall along the marginal zone as well as through the intermediate zone in a presumed radial glial independent manner, using corticofugal fibers as substrates [5]–[15], although a functional relationship between radial glia and migrating interneurons has also been suggested [16]. A subset of these interneurons navigate radially inwards toward the ventricular zone before turning back to migrate towards the cortical plate [11], [12]. A diverse set of molecular cues, such as Dlx1/2 transcription factor regulated signals, motogenic factors such as BDNF, NT-4, and HGF, chemoattractants such as GDNF and NRG-1, as well as members of semaphorin family (3A, F) of chemorepellent factors, modulate the navigation of interneurons from the ganglionic eminence into the cerebral wall [1].

How interneurons migrate and position themselves in the appropriate laminar and areal locations can influence the patterns of their connectivity and function. The altered function of GABAergic interneurons in the forebrain is thought to contribute to the cognitive functional deficits in neurodevelopmental disorders such as schizophrenia [reviewed in 17], [18]. In spite of its significance, due to the lack of live imaging of genetically defined populations of interneurons and radial glia, little is known about the inter-cellular mechanisms that influence the movement and placement of interneurons within the developing cerebral wall and how distinct modes of interneuronal movement are coordinated within the cortex.

To investigate the mechanisms of interneuronal movement within the developing cerebral wall and determine, what, if any influence, the radial glial grid exerts on the navigation of interneurons within the developing cerebral cortex, we live imaged the migration of genetically defined population of interneurons and their interactions with the radial glial scaffold in the developing cerebral wall. We used *Dlx5/6-cre-IRES-EGFP* (*Dlx5/6-CIE*) mice in which only interneurons generated from the ganglionic eminence express EGFP [19]. Radial glia specific BLBP promoter-DsRed2 DNA was electroporated into *Dlx5/6-CIE* mice cortices to label radial glia with DsRed2. Interneuronal migration was also monitored *in utero* in developing embryos using two-photon microscopy. These studies reveal potentially significant radial glial influence on tangential migration of interneurons and show that interneuronal movement in pallial to subpallial direction, occurring all across the cerebral wall, may contribute to the appropriate positioning of interneurons within the developing cerebral cortex.

## Results

### Interneuron-radial glial interactions across the developing cerebral wall

**Figure 1:**
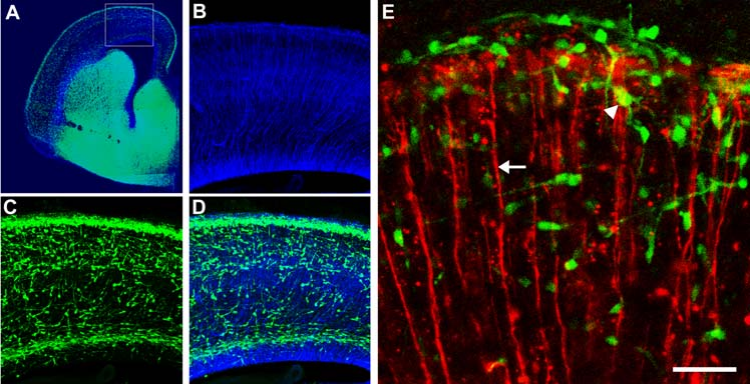
Analysis of interneuron-radial glial interactions in the embryonic cerebral cortex. (A) Interneurons express GFP in a coronal slice of E16 cortex from Dlx5/6-cre-IRES-EGFP (Dlx5/6-CIE) mice. Radial glial scaffold is labeled with RC2 antibodies (blue). (B–D) Higher magnification view of the outlined region (B, radial glial scaffold; C, GFP^+^ interneurons, D, merge) illustrates the potential for interneuron-radial glial interactions as the neurons invade the cerebral wall. (E) Assay for live imaging of radial glia-interneuronal interactions. Dlx5/6-CIE E16 cerebral cortices were electroporated with BLBP-DsRed2 to label multiple radial glia. Time-lapse imaging of slices from these electroporated cortices were used to evaluate interneuron [arrowhead]-radial glial [arrow] interactions. GE, ganglionic eminence; CP, cortical plate; IZ, intermediate zone; VZ, ventricular zone. Scale bar: A, 1250 µm; B–D, 350 µm; E, 80 µm.

**Figure 2:**
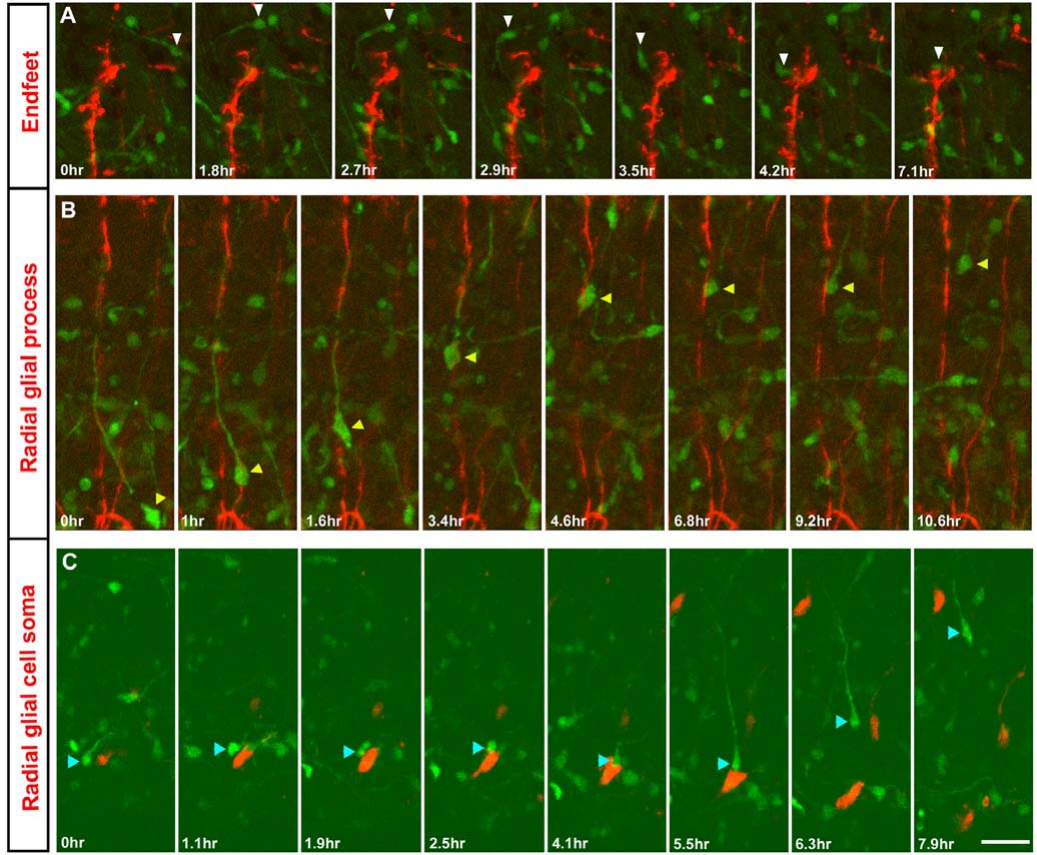
Radial glia modulates the patterns of interneuronal migration. (A) An interneuron [arrowhead] migrating in the marginal zone area turn towards a radial glial endfeet [red]. (B) An interneuron [arrowhead] ascending towards the cortical plate uses radial glial strand [red] as a migratory guide, before detaching from it. (C) Contact with a radial glial cell soma [red] in the ventricular zone alters the trajectory of an interneuron [arrowhead] from tangential to sharply radial. Time elapsed since the beginning of observations is indicated in minutes. (Also, see the AVI movie files provided in the supporting information). Scale bar: A, C, 100 µm; B, 80 µm.

**Figure 3:**
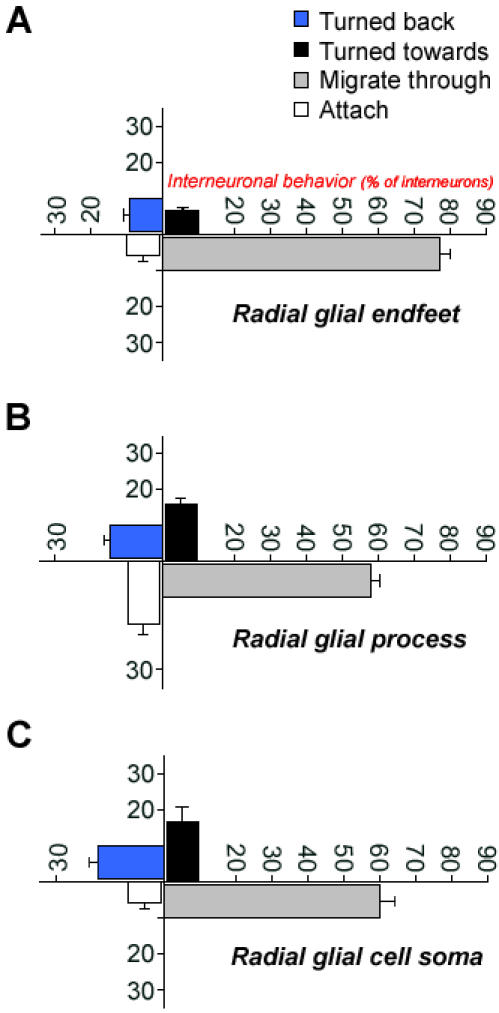
Changes in interneuronal migration following interactions with radial glia. (A–C) Changes in interneuronal migratory behavior before and after contact with radial glial endfeet (A), process (B), and cell soma (C) were monitored in real time. Interneurons displayed one of the following four types of behavior when they come in contact with a radial glial cell: (1) turn towards the radial glial cell, (2) migrate pass the radial glia without pausing, (3) turn back and migrate in opposite direction after contacting radial glia, or (4) remain attached to the radial glial cell following contact. The extent of these four types of interactions were measured per radial glial endfeet, process, or cell soma during the period of observation. Distinct radial glial cells can alter the migratory behavior of subsets of interneurons in the developing cerebral wall. Number of cells/group>1000. Data shown are mean±SEM.

To investigate how the movement of interneurons occurs within the radial glial scaffold of the developing cerebral cortex, we labeled radial glia in *Dlx5/6-CIE* mice with BLBP promoter-DsRed2 electroporation and live imaged interneuron (GFP^+^)-radial glial (DsRed2^+^) interactions (Fig. 1). This approach labels multiple, but not all, radial glia, thus enabling the evaluation of individual interneuron-radial glial interactions. Interneurons migrating along the superficial surface of the developing cortex, through middle of the cerebral wall, and in the ventricular zone (VZ) displayed strikingly distinct patterns of interactions with radial glia. Interneurons migrating in the marginal zone area migrate through a dynamically active `sieve' of radial glial endfeet (Fig. 2A, Fig. 3A, Fig. S1, Movies S1, S1a and S2). As they migrate, these neurons are contacted by probing radial glial endfeet branches. Interneuronal behavior was monitored before and after contact with individual radial glial cells. Four types of interactions were noticed: First, some interneurons (11.2±1.4%) turn back after radial glial contact. A second subset of interneurons (73.0±2.7%) migrate uninterrupted further dorso-medially into the cerebral wall despite of probing radial glial contacts. 7.7±1.2% of interneurons pause as glial endfeet branches contacted them. These pausing neurons eventually migrate through or turn back. Finally, 8.0±1.0% of interneurons that come in contact with radial glial endfeet turn radially inwards into the cortical plate (Fig. 2A, Fig. 3A, Movies S1 and S2). Radial glia endfeet interacting with this subset of interneurons appear to facilitate the turning of these neurons inwards towards the emerging cortical plate (CP). These observations suggest that dynamically active radial glial endfeet can critically influence the migratory behavior of distinct subsets of interneurons.

Interneurons moving tangentially in the intermediate zone (IZ) region are thought to migrate along axonal processes without any apparent interactions with radial glia [1]. Although radially oriented interneurons in the cortical plate make multiple contacts with radial glia, the orientation of the leading processes of these cells are always not aligned with radial glia processes in fixed tissue preparations, leading to the suggestion that these GE derived interneurons probably do not migrate along radial glia [16]. However, live imaging of neuron-radial glial interactions indicates that these neurons actively probe different radial glial strands during their tangentially oriented migration (Fig. 2B, Fig. 3B, Movies S3, S4, S5, S6, S7). Many neurons (54.7±2.9%) continue to migrate uninterrupted after contacting perpendicularly oriented radial glial strands, whereas others turn back and reorient their migration in the opposite direction (14.7±1.7%) or pause (16.2±2.4%) after coming in contact with specific radial glial strands (Movies S3, S4, S5, S6, S7). Subsets of interneurons (13.9±1.6%) can also change their mode of translocation from tangential to radial following contacts with specific radial glia strands (Fig. 3B). These radially migrating interneurons make extensive contacts with radial glial processes as they migrate up towards the CP (Movies S3, S4, and S8). Furthermore, interneurons also migrate radially inwards past the cortical plate towards the ventricular zone prior to turning back to journey towards their final laminar positions in the CP. The neurons that dive in towards the VZ contact, probe, and glide along distinct radial glial strands during their downward movement (Movies S4, S5).

In the ventricular zone, tangentially migrating interneurons often contact radial glial cell soma (Fig. 2C, Fig. 3C, Movies S8, S9, S10, S11, S12). The direction of subsets of interneurons (18.3±4.1%) in the VZ changes from tangential to sharply radial upon contact with radial glial cell soma (Movies S9, S10). This directional change can occur when interneurons contact either an undividing or an apparently asymmetrically dividing radial glial cell (Movies S9, S10). Furthermore, 57.0±3.8% of interneurons that come in contact with a radial glial cell soma continued their migration uninterrupted, whereas 19.2±2.4% of neurons turned back and migrated tangentially in the opposite direction (Movie S12). Together, these observations suggest that radial glial scaffold can critically influence interneuronal migration and placement in the developing cerebral cortex by attracting, repulsing, stalling, or permitting the continued migration of distinct subsets of interneurons.

### Local migration of interneurons within the developing cerebral wall

**Figure 4:**
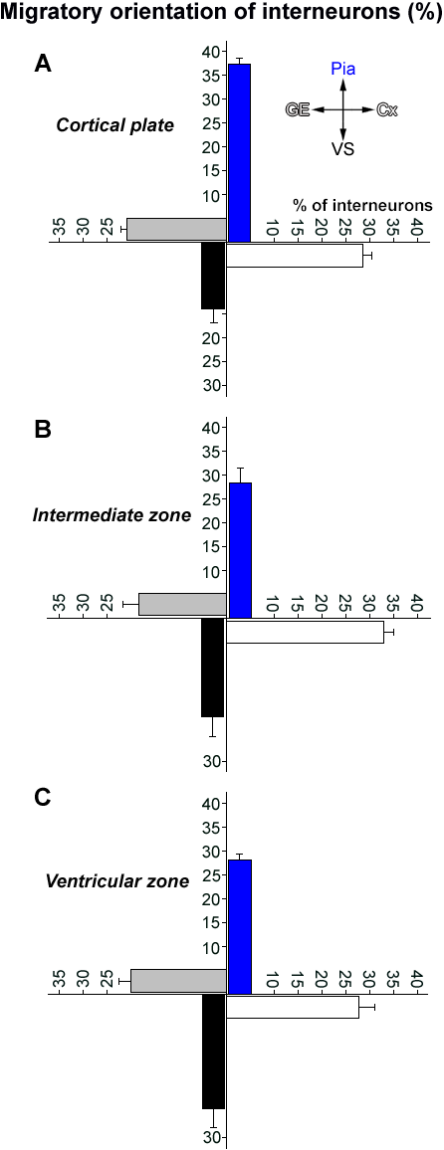
Quantification of the direction of interneuronal migration in the embryonic cortex. (A–C) Neurons migrating in four distinct directions (towards the pial surface, towards the ventricular surface, tangentially towards cortex [subpallium→ pallium], tangentially towards ganglionic eminence [pallium→ subpallium]) within the cortical plate (A), intermediate zone (B), and ventricular zone (C) were measured. Surprisingly, significant numbers of interneurons across the cerebral wall migrate in cortex→ ganglionic eminence direction. Number of cells/group>1500. Data shown are mean±SEM.

**Figure 5 pone-0000794-g005:**
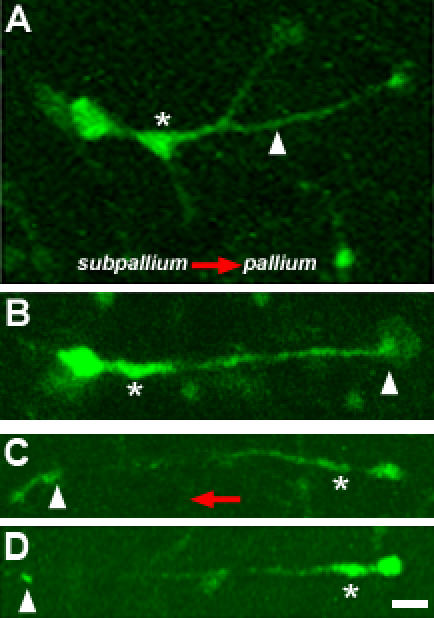
Distinct migratory dynamics of tangentially migrating interneurons. Interneurons migrating tangentially in subpallium → pallium orientation extend branched leading processes (arrowhead, A) with elaborate growth cones (arrowhead, B) trailed by pre-somal (asterisk, A, B) swellings. (C, D) The leading processes of interneurons migrating in the opposite direction [pallium→ subpallium] tend not to branch and their tips are often club-like and small (arrowhead, C, D). Pre-somal swellings are evident in these neurons (asterisk, C, D). These panels are freeze frame images of actively migrating neurons. Scale bar: 20 µm.

Aside from delineating novel dynamic interactions between radial glia and interneurons, live imaging of entire populations of interneurons in embryonic cerebral cortex also revealed several distinct aspects of interneuronal migration. E16 *Dlx5/6-CIE* cerebral cortices were vibratome sliced and interneuronal movement in these cortices were immediately imaged. Though the previously described [1], [20] general patterns of tangential interneuronal movement from subpallium towards pallium in a lateral to medial direction is evident across the cerebral wall, surprisingly, the interneuronal movement is considerably bidirectional, with a significant number of them moving in the opposite direction-i.e., mediolaterally in pallial to subpallial direction in all regions of the cerebral wall (Movies S13, S14). 20.0±2.4%, 18.3±3.3%, and 20.7±1.4% of interneurons migrate in this manner in the ventricular zone, intermediate zone, and cortical plate regions of the cerebral wall, respectively (Fig. 4, Movies S15, S16, S17, S18, S19, S20, S21, S22 [CP: Movies S15, S16, S17, S18; IZ: Movie S19; VZ: Movies S20, S21, S22]). Furthermore, interneurons migrating from subpallium to pallium display stereotypic migratory behavior: elongated, probing, branched leading processes trailed by pre-somal swellings into which nucleus and cell soma translocates while retracting the trailing process [16], [21]–[23]. Compared to their counterparts migrating in subpallial to pallial direction, 47.0±7.0% fewer of the neurons that migrate in the opposite direction displayed this characteristic interneuronal migratory dynamics. The leading processes of these neurons tend not to branch and remain club-like as they migrate (Fig. 5). The rate of migration of both of these populations of tangentially migrating interneurons is similar in the cortical plate (subpallial to pallial oriented interneurons, 40.0±5.9 µm/hr [n = 30]; pallial to subpallial oriented interneurons, 46.1±5.7 µm/hr [n = 30]) and ventricular zone (subpallial to pallial oriented interneurons, 51.0±6.6 µm/hr [n = 30]; pallial to subpallial oriented interneurons, 62.0±12.0 µm/hr [n = 30]). However, in the intermediate zone, the rate of migration of neurons that are migrating in subpallial orientation is significantly lower (36.0±6.6 µm/hr [n = 30]) when compared to the ones migrating towards pallium (53.8±1.4 µm/hr [n = 30]; P<0.01, Student's *t*-test). These results indicate that pallial to subpallial oriented migration of interneurons is distinctly different in its dynamics than the previously described patterns of interneuronal migration. The prevalence of this mode of migration all across the cerebral wall, in the VZ, IZ, and CP, suggest that this type of migration may significantly influence how interneurons achieve their positioning within the developing cortex.

### Cellular diversity of interneuronal migratory behavior

**Figure 6 pone-0000794-g006:**
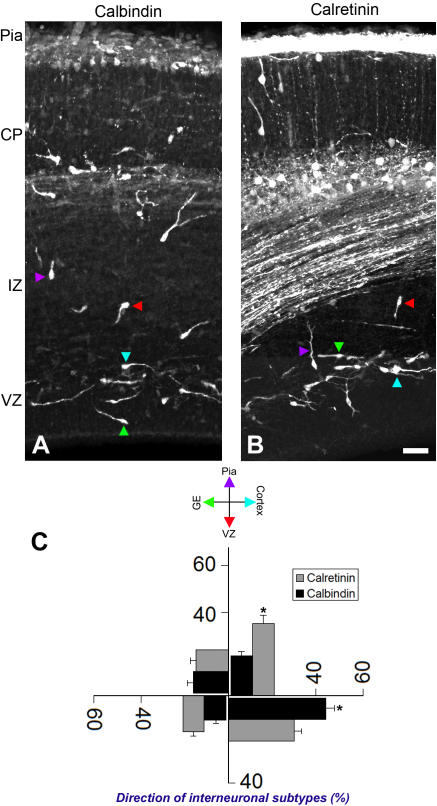
Migratory dynamics of distinct subsets of interneurons. (A, B) Interneurons in embryonic day 16 cerebral cortex were labeled with anti-calbindin or calretinin antibodies. (C) The migratory orientation of labeled interneurons as indicated by the direction of their leading processes was quantified across the cerebral wall. Majority of calretinin^+^ cells were oriented radially, towards pial surface direction, whereas a higher percentage of calbindin^+^ interneurons were oriented tangentially towards the cortex. Distinct subsets of interneurons may therefore undergo distinct patterns of migration within the cerebral wall. CP, cortical plate; IZ, intermediate zone; VZ, ventricular zone. Data shown are mean±SEM (n = 10); asterisk, significant when compared with controls at p<0.01 (Student's t test). Scale bar: 75 µm.

Since specific areal and laminar positioning of different types of interneurons within the cerebral cortex suggests that subclasses of interneurons may engage in different modes of migration to reach their target locations, we investigated if potentially different types of interneurons display any differences in their oriented migration. We immunolabelled embryonic cortex (E16) with interneuron specific anti-calretinin or calbindin antibodies and analyzed the orientation of the leading processes of these neurons. The leading process of a migrating interneuron is oriented towards the direction of migration and thus provides an index of orientation of migration [24]–[26]. Compared to calbindin^+^ interneurons, significantly higher number of calretinin^+^ cells were oriented radially, towards pial surface direction (20.9±2.9% vs 34.4±4.0%; Fig. 6). However, significantly higher percentage of calbindin^+^ interneurons were oriented towards the dorsal cortex (46.0±3.1% vs 31.1±3.6%; Fig. 6). Together, these observations suggest that distinct types of interneurons, as recognized by the differential expression of neurochemical markers such as calbindin or calretinin, may engage in distinct patterns of migration to navigate their way to their targets.

### Distinct motile behavior of interneurons within the ventricular zone

As noted previously, the extent of interneuronal migration towards the ventricular zone also appears to be significant [10], [11], [19]. The percentage of interneurons moving in the direction of the ventricular surface in the cortical plate, intermediate zone, and ventricular zone are 13.7±2.8%, 20.6±4.0%, 24.3±3.8%, respectively (Fig. 4). It was suggested that in the VZ, these neurons extend a leading process to contact the ventricular surface, retract the process, and migrate back towards the CP [11], [12], [20]. However, the behavior of these neurons appears to be more dynamic and complex. The elongated leading process of these ventricle directed neurons initially scans the ventricular surface. These neurons then often sprout multiple processes as they move tangentially at the bottom of the VZ, prior to migrating up radially towards CP. A subset of these neurons continues to migrate in tangential direction within the VZ or after first migrating up into the IZ (Movies S20, S21, S22).

### Interneuronal migration in the intact embryonic brain

**Figure 7:**
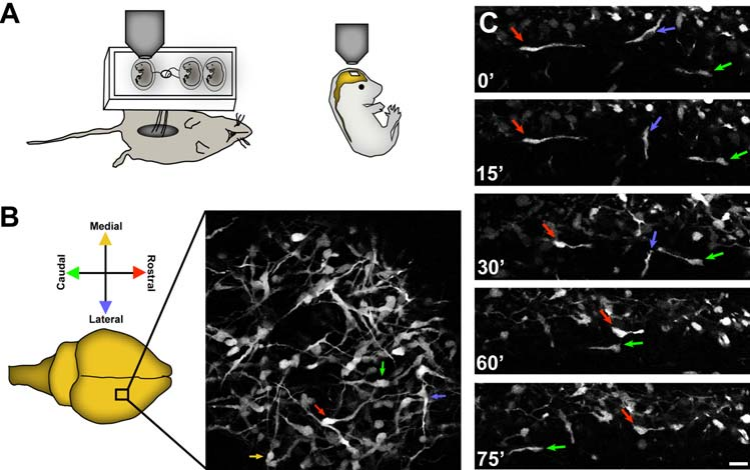
In vivo two-photon microscopy of interneurons in living embryos. (A) Living embryos (E15) attached to the mother were immobilized on an optical stage. Two-photon imaging of GFP positive interneurons in the superficial surface of parietal cortical area (B) indicates multidirectional orientation of migrating interneurons. (C) Time lapse imaging of these neurons illustrate multidirectional movement of these neurons as they navigate to their appropriate locations within the developing cortex. This multidirectional movement of interneurons suggest that once interneurons invade cortex from the ganglionic eminence, local cues may influence the allocation of interneurons into distinct cortical areas. Arrows indicate neurons moving in different orientations. Time elapsed is indicated in minutes. M, medial; L, lateral; R, rostral; C, caudal. Scale bar: B, 30 µm; C, 40 µm.

As the interneurons ascend to the cortical plate, many of them end their migration at the top of the developing cortex. However, 12.8±1.5% (n = 311) of the interneurons migrate through to the top of the cortical plate, turn laterally in a tangential direction, and migrate along the outermost layer of the developing cortex to a different cortical region (Movie S18). These results suggest that there is considerable local interneuronal movement within the developing cerebral wall. To investigate the local migration of interneurons in the developing cerebral cortex further and to determine if multidirectional interneuronal movement occurs in the emerging neocortex of intact, living embryos, we live imaged interneurons *in utero* using multiphoton microscopy. Mediolateral region of the forebrain of embryonic day15 embryos was imaged *in utero* while they remained attached to the mother. Interneurons migrated in multiple orientations. In addition to the previously known lateral-medial directed migration, interneurons also migrated in medial-lateral as well as rostrocaudal directions (Fig. 7). This multi-directional migration within local areas of the developing cortex may facilitate the appropriate placement of interneurons within distinct cortical regions.

## Discussion

The live imaging of genetically defined populations of interneurons and radial glia in the developing cerebral cortex suggests that interneuronal positioning in the developing cerebral cortex may involve significant interactions with radial glia and multi-directional local migration, especially in dorsal pallium to subpallium direction all across the cerebral wall. In contrast to previously suggested model of cortical neuronal migrations in which pyramidal neurons migrate in a radial glial dependent manner and tangentially migrating interneurons migrate mainly in a radial glial independent manner [1], [12], these live imaging observations are consistent with the possibility that radial glial cells can repulse, attract, or allow oriented migration of interneuronal subsets in the developing cortex.

Our observations (Fig. 4, Movies S13 and S14) and previous time lapse analysis of vital dye or GAD67-GFP labeled neurons in the marginal zone indicate that marginal zone interneurons can migrate in multiple directions [22], [27]. Once these interneurons migrating in the marginal zone reach their areal destinations, they are thought to descend into the underlying cortex to assume their appropriate positions [22]. How this is orchestrated remains to be fully delineated, though superficial Cajal- Retzius neurons or pyramidal projection neurons were speculated to play a role [16], [22], [28], [29]. Our observations suggest local radial glial cells, by their potential ability to repulse, attract, stall, or permit the continued migration of distinct subsets of interneurons, may play an essential role in the targeting of appropriate subsets of interneurons into appropriate areal locations (Fig. 2, 3, Movies S1, S2). In a ferret model of cortical dysplasia, induced with an antimitotic agent, methylazoxy methanol (MAM), radial glial scaffold is disrupted and GE derived interneurons do not migrate properly into the cortical plate [30]. In mutant mice with radial glial endfeet abnormalities (β1 or α6 integrin, perlecan, FAK, and laminin γ nidogen binding site mutants [31]–[33]), radial glial proliferation, interkinetic nuclear movement of radial glia, and neurogenesis appears to be unaffected, but significant neuronal placement deficits are evident [34]. Considering the role the dynamic activity of radial glial endfeet play in interneuronal migration, neuronal positioning deficits noticed in these mutant mice , in part, may have resulted from altered radial glial endfeet-migrating neuron interactions. The relative significance and identity of secreted or surface bound radial glial signals during these interactions and how and if they change the interneuronal adhesive interactions with corticofugal fibers, the other known substrate of interneuronal migration in cortex [8], [10], remains unclear. Furthermore, radial glia endfeet may also function simply as a physical obstruction to cause a subset of interneurons to alter their trajectory. How frequently interneurons can change their course of migration without contact with endfeet also remains to be characterized. Further live-imaging analysis of interneuronal migration in mutant mice with radial glial cell deficits and in preparations in which the entire population of radial glia can be visualized, will be essential to fully establish the significance of interneuron-radial glia interactions in the modulation of interneuron migration dynamics.

The multimodal migratory behavior of interneurons, especially the movement of interneurons in tangential, mediolateral direction towards the subpallium within the cerebral wall (Figures 4–7) and along the marginal zone or ventricular surface [27], may simply be a result of transient, explorative migration in mediolateral direction before resumption of forward movement. Alternatively, it is conceivable that interneurons, in addition to responding to gradients of attractants within the developing dorsal pallium [35], may also respond to more localized, counter guidance cues within specific areas of the cerebral wall. Such local signals may enable distinct classes of interneurons to navigate to their appropriate areal locations within the developing cerebral cortex. Furthermore, the extent and dynamics of changes in interneuronal migration in the ventricular zone suggest that interneurons may potentially influence radial glial proliferative events in the VZ and may themselves receive information regarding their areal position and radial trajectory (Fig. 2, 3, Movies S8, S9, S10, S11, S12, S20, S21, S22). Recent studies by Sawamoto et al. [36] suggest that the ciliated ependymal cells of the lateral ventricles may be essential in conveying gradients of chemoguidance cues from the ventricles to migrating neuroblasts in the VZ. Interneurons probing the ventricular surface may receive and process similar directional or positional cues as they navigate to their laminar positions in distinct cortical areas. The distinct migration history of interneurons within the developing cerebral wall may guide and define interneuronal diversity in cerebral cortex. Real time imaging analysis using interneuron specific gene deletion models will help to clarify this possibility.

**Figure 8:**
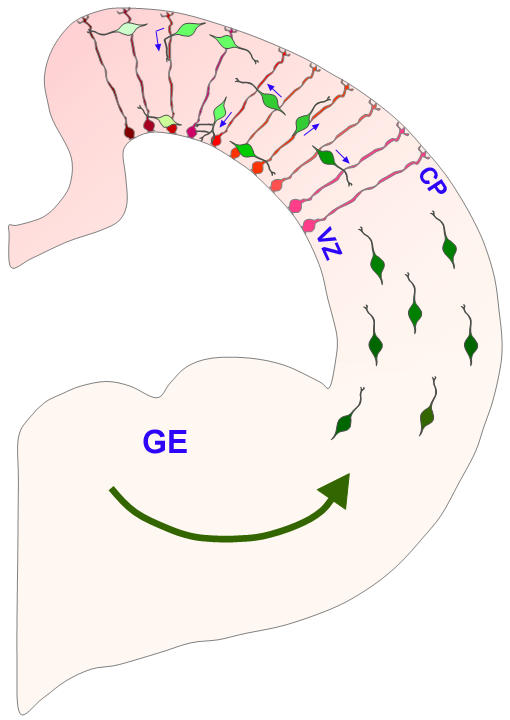
Interneuron-radial glial interactions in the developing cerebral cortex. Interneurons (green) migrating into the cerebral wall from the ganglionic eminence(GE) interact with radial glia (red) and can exhibit changes in direction of migration after contacting radial glia. Interneurons can use radial glia as a scaffold upon which to migrate as they ascend to the cortical plate (CP) or descend in the direction of the ventricular zone (VZ). Particular orientation and morphological dynamics of migration may be associated with particular subsets of interneurons. Once the interneurons invade the cortex from the ganglionic eminence, differential interactions between interneurons and radial glial scaffold and localized multidirectional migration of interneurons influenced by local guidance cues may facilitate interneuronal positioning within distinct domains of the developing cerebral cortex. Potential differences among interneurons and radial glia are indicated by shades of green and red, respectively. Putative local guidance cues are indicated by a gradient of pink. Blue arrows indicate direction of migration.

Physiological and morphological characterization of interneurons indicate that cortical interneurons as a group is highly diverse. Transcription factor expression analysis of ganglionic eminence progenitor domains suggests that distinct progenitor domains in subpallium may give rise to distinct types of cortical interneurons [37]–[46]. Distinct subtypes of interneurons display different migratory dynamics (Fig. 5, 6). Similarly, distinct areal and molecular differences are also evident in developing cortical radial glial cells [47]. The nature of interactions between radial glia and interneurons suggests that distinct groups of migrating interneurons are influenced differentially by the radial glial scaffold within the developing cortex. Since only a subset of migrating interneurons alter their behavior following interactions with a particular radial glial cell (Fig. 2, 3), there might be specific positional affinities between distinct interneurons and radial glia across the developing cerebral cortex. Once the interneurons invade the cortex from the ganglionic eminence, differential interactions between interneurons and areally distinct radial glial scaffold may alter the migratory trajectory of interneurons, and thus facilitate interneuronal positioning within distinct areal and laminar domains of the developing cerebral cortex (Fig. 8). Though the nature of signaling mechanisms utilized by the radial glial cells to influence the migration of interneurons remains to be fully elucidated, the ability of radial glia to influence both the radial and tangential migration of neurons suggest that radial glia may provide a structural and molecular cue matrix to coordinate the placement of both interneurons and pyramidal neurons in the developing cerebral cortex.

## Materials and Methods

### Analysis of radial glia-interneuronal interactions

Lateral ventricles of embryonic day 16 Dlx5/6-cre-IRES-EGFP mouse embryos were injected with 2.5 µl of a plasmid mixture, containing 3 µg/µl pBLBP-DsRed2 DNA diluted 1∶1 with mouse neuron nucleofector solution (Amaxa biosystems)/0.001% fast green, and subjected to electroporation as described previously [48]. Following electroporation, cortices were removed from the embryos, coronally sectioned (200 µm) in a vibratome (Leica VT 1000S), mounted on nucleopore membrane filters, and maintained in MEM/10% FBS at 37°C/5% CO_2_. Within 24 hours, GFP labeled interneurons and DsRed2 tagged radial glia spanning the cerebral wall in the medio-dorsal region of the cerebral cortex were repeatedly imaged, using a 20× objective, in a Zeiss inverted microscope attached to a PASCAL confocal laser scanning system and a live cell incubation chamber. An argon ion laser at 488nm and a HeNe laser at 543nm were used for excitation of GFP and DsRed2, respectively. Labeling and imaging of multiple, individual radial glial cells with this approach in *Dlx5/6-CIE* mice enables the real time analysis of radial glia-interneuron interactions all across the developing cerebral wall. Interneurons turned toward, migrated pass, turned back, or attached to radial glial cells when they interacted with them. Changes in the migratory behavior of interneurons after they come in contact with each radial glial endfeet, process, or cell soma were counted during the period of time-lapse observation.

To study different modes of interneuronal migration, E16 cerebral cortices from *Dlx5/6-CIE* mice were vibratome sliced, and soon after sectioning, interneuronal movement was repeatedly imaged for up to 14 hours. The rate of migration of neurons and the orientation of migration of neurons within regions of the cerebral wall were measured as described previously [24], [49], [50]. To quantify the number of interneurons migrating between cortical areas, a 25000 µm^2^ box was positioned at the top of the cortical plate, and the percentage of neurons within this area that migrated to the top of the cortex, turn laterally, and migrated to a different cortical region was measured during the period of time-lapse observation. All quantifications of radial glial, interneuronal dynamics were made using the Zeiss Pascal, or LSM510 software. Statistical differences between experimental groups were tested by Student's *t* test.

### *In utero* two photon microscopy of interneuronal migration

Pregnant (E15) *Dlx5/6-CIE* mice were anesthetized using avertin (0.2 ml/10 g body weight), placed on a specially designed optical stage, embryos in the opened uterine sac were positioned so that the mediolateral areas of the forebrain were pointing upwards, and the embryos were then immobilized with a mix of artificial cerebrospinal fluid and 6% low melting point agarose within a container attached to the optical stage. The optical stage containing the embryos and mother was attached to a Zeiss LSM510 multiphoton microscope and *in vivo* images of GFP expressing interneurons from the superficial surfaces of mediolateral regions of the forebrain were repeatedly acquired using a 40× water immersion objective, every 5–10 minutes, for ∼2–4 hours. A Zeiss LSM 510 NLO multiphoton system with a Mira 900 IR laser tuned at 860–870nm was used for excitation of GFP.

### Immunohistochemistry

E16 embryonic cortices were vibratome sectioned and immunolabelled [24] with one of the following primary antibodies: RC2 (Iowa Hybridoma Bank), Anti-GABA (Santa Cruz Biotechnology; Cytoskeleton Inc.), anti-GAD (Santa Cruz Biotechnology), anti-calretinin (Chemicon), anti-calbindin (Chemicon), and Tuj-1 (Covance). Immunoreactivity was detected with Cy2 or Cy3 conjugated anti-mouse or anti-rabbit secondary antibodies (Jackson Immunoresearch).

## Supporting Information

Figure S1 Radial glia-interneuron contact. Direct association between radial glia endfeet and migrating neurons shown in supplementary movie S1 is evident in X or Y axis line scans through a single optical plane. White arrow in the central panel indicates a neuron contacting a branch of radial glial endfeet. In top and right side of the panel, line scans of this region show interneuron (green) and radial glia endfeet (red) contact (arrow) on the same plane.
(1.82 MB TIF)

Movie S1 Interactions between radial glial endfeet (red) and migrating interneurons (green). Arrowhead points to the area of interest. Radial glial endfeet appear to repulse, stall, or permit the continued migration of distinct subsets of interneurons. Asterisk points to a neuron that turns back after contact with radial glia. Time length = 9.4 hrs.
(1.42 MB MOV)

Movie S1a 3D rotation of a single time frame from supplementary movie1 shows positions of migrating neurons and radial glia end feet on the same plane. Also see Figure S1.
(1.02 MB AVI)

Movie S2 Interneuron (arrowhead) turning towards radial glial end feet (red). Time length = 9.1 hrs.
(0.62 MB MOV)

Movie S3 Radial glial process-interneuron interactions. An ascending interneuron (arrowhead) in the intermediate zone uses a radial glial process (red) as a migratory guide, before detaching from it. Time length = 12.2 hrs.
(0.96 MB MOV)

Movie S4 Radial glial process-interneuron interactions. An inwardly migrating interneuron (yellow arrowhead) briefly associates with a radial glial strand (red) as an upwardly migrating interneuron (white arrowhead) use the same glial strand to ascend towards the cortical plate. Time length = 5.2 hrs.
(0.60 MB MOV)

Movie S5 An inwardly migrating interneuron (arrowhead) in the intermediate zone probes and associates with multiple radial glial processes (red) as it migrates towards the ventricular surface. Time length = 10.0 hrs.
(0.80 MB MOV)

Movie S6 Interactions between a radial glial process (red) and a tangentially migrating interneuron (arrowhead) in the intermediate zone. This neuron turns away after coming in contact with this radial glial strand. Time length = 3.6 hrs.
(0.35 MB MOV)

Movie S7 Interactions between radial glial processes (red) and interneurons (asterisks). These neurons (asterisks) slowdown or pause as they interact with the radial glial strands. Time length = 7.2 hrs.
(0.59 MB MOV)

Movie S8 Interactions between a radial glial cell and an interneuron in the VZ. This neuron (arrowhead) attaches to a radial glial cell (red) and ascends up in close apposition to the radial glial strand. The radial glia cell soma also moves up slightly. Time length = 3.0 hrs.
(0.15 MB MOV)

Movie S9 Interactions between radial glial soma and an interneuron in the VZ. The interneuron (arrowhead) changes its trajectory from tangential to radial after contacting the radial glial cell soma (red). Time length = 9.3 hrs.
(0.57 MB MOV)

Movie S10 Interactions between radial glial soma and an interneuron in the VZ. This interneuron (arrowhead) changes its trajectory from tangential to radial after contacting the radial glial cell (red), which then appear to divide asymmetrically (parallel to the direction of the ventricular surface). Time length = 6.5 hrs.
(0.67 MB MOV)

Movie S11 Interactions between a radial glial cell and an interneuron in the VZ. This interneuron (arrowhead) change its trajectory after contacting the radial glial cell (red) and begin to climb along the radial glial process. Time length = 9.0 hrs.
(0.50 MB MOV)

Movie S12 Interactions between a radial glial and an interneuron in the VZ. This interneuron (asterisk) turns back after contacting the radial glial cell soma (red). Time length = 4.3 hrs.
(0.29 MB MOV)

Movie S13 Migration of interneurons in the embryonic cerebral cortex. E16 cerebral cortex from Dlx5/6-CIE mice were repeatedly imaged at 8 minute intervals using a Zeiss inverted microscope attached to a PASCAL confocal laser scanning system and a live cell incubation chamber. These images were compiled as AVI movie files to illustrate the extent of different modes of interneuronal migration in the developing cerebral wall. Most of the interneurons migrate tangentially from the ganglionic eminence towards cortex and in radial directions towards the cortical plate or ventricular surface. A significant number of interneurons also migrate in cortex → ganglionic eminence direction. White arrows point to general direction of different cerebral cortical regions. GE, ganglionic eminence; CX, cortex; VS, ventricular surface. Time length = 11.2 hrs.
(1.19 MB MOV)

Movie S14 Migration of interneurons in the embryonic cerebral cortex. Another example of different modes of interneuronal migration in the E16 cerebral cortex. Most of the interneurons migrate tangentially from the ganglionic eminence towards cortex and in radial directions towards the cortical plate or ventricular surface. A significant number of interneurons also migrate in cortex → ganglionic eminence direction. White arrows point to general direction of different cerebral cortical regions. GE, ganglionic eminence; CX, cortex; VS, ventricular surface. Time length = 11.2 hrs.
(1.37 MB MOV)

Movie S15 Multi directional interneuronal migration in the cortical plate. Time length = 5.2 hrs.
(0.70 MB MOV)

Movie S16 Multi directional interneuronal migration in the cortical plate. Different colored asterisks indicate neurons migrating in distinct orientations. Time length = 6.5 hrs
(0.71 MB MOV)

Movie S17 Multi directional interneuronal migration in the cortical plate. Different colored asterisks indicate neurons migrating in distinct orientations. Time length = 7.4 hrs
(0.87 MB MOV)

Movie S18 Dynamics of interneuronal migration in the cortical plate. Asterisk points to an interneuron that migrated to the top of the cortex, turned lateral along the marginal zone into a different cortical area, and then turned back. Time length = 14.0 hrs.
(0.86 MB MOV)

Movie S19 Interneuronal migration in the intermediate zone. Different colored asterisks indicate neurons migrating in distinct orientations. Time length = 11.2 hrs.
(1.41 MB MOV)

Movie S20 Interneuronal migration in the ventricular zone. Time length = 11.2 hrs.
(1.27 MB MOV)

Movie S21 Interneuronal migration in the ventricular zone. Time length = 5.0 hrs.
(0.68 MB MOV)

Movie S22 Dynamics of interneuronal migration in the ventricular zone. An inwardly migrating interneuron (arrowhead) contacts, branches, and scans the ventricular surface prior to migrating tangentially within the VZ. Time length = 3.5 hrs.
(0.68 MB MOV)
