# Supplementary figures and images for: Radial Glial Dependent and Independent Dynamics of Interneuronal Migration in the Developing Cerebral Cortex
Source: PLoS One. 2007 Aug 29;2(8):e794. doi: 10.1371/journal.pone.0000794 (PMC1950908; doi:10.1371/journal.pone.0000794)

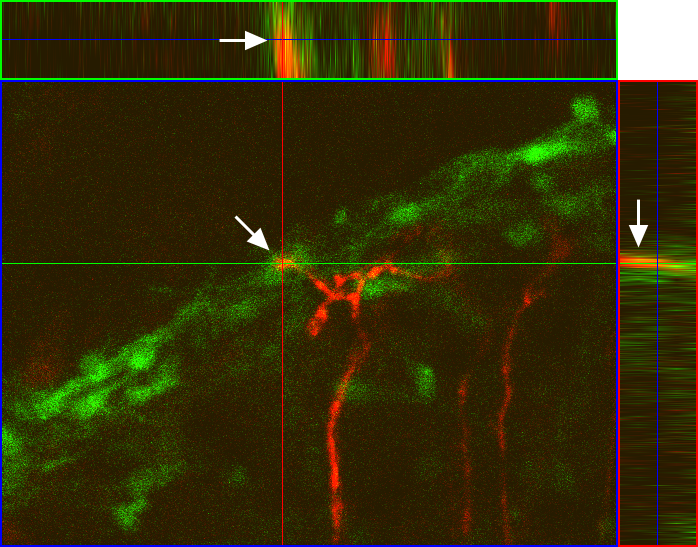

Supplement: Figure S1 — Radial glia-interneuron contact. Direct association between radial glia endfeet and migrating neurons shown in supplementary movie S1 is evident in X or Y axis line scans through a single optical plane. White arrow in the central panel indicates a neuron contacting a branch of radial glial endfeet. In top and right side of the panel, line scans of this region show interneuron (green) and radial glia endfeet (red) contact (arrow) on the same plane. (1.82 MB TIF) [file pone.0000794.s001.tif]
